# The brain mechanism of awakening dysfunction in children with primary nocturnal enuresis based on PVT-NAc neural pathway: a resting-state fMRI study

**DOI:** 10.1038/s41598-021-96519-w

**Published:** 2021-08-24

**Authors:** Kaihua Jiang, Peng Xue, Yue Xu, Yang Yi, Jie Zhu, Li Ding, Aibin Zheng

**Affiliations:** Department of Pediatrics, Changzhou Children’s Hospital of Nantong University, No. 468 Yanling Road, Changzhou, 213003 Jiangsu People’s Republic of China

**Keywords:** Neurology, Urology

## Abstract

Primary nocturnal enuresis (PNE) affects children’s physical and mental health with a high rate. However, its neural mechanism is still unclear. Studies have found that the paraventricular thalamus (PVT) is among the key brain regions implicated with awakening regulation and its control of the transition between sleep and wakening is dependent on signaling through the PVT-nucleus accumbens (NAc) pathway. So this study analyzed the function of brain regions and their connectivity of PVT and NAc. A total of twenty-six PNE and typically developing (TD) children were involved in the study and the methods of amplitude of low frequency fluctuation (ALFF), degree centrality (DC) and functional connectivity (FC) based on resting-state functional magnetic resonance imaging (rs-fMRI) were used to analyze the brain functions. Results showed that there was no statistical significant difference in ALFF and DC between PNE and TD children in bilateral PVT and NAc. And there was statistical significant difference of the comparison of the FC of left PVT (lPVT) and left NAc (lNAc) between PNE and TD children. Meanwhile, there was negative correlation between awakening score and the FC of rPVT and lNAc, and no obvious correlation between awakening score and the FC of lPVT and lNAc in PNE children. Meanwhile, there was both negative correlation between awakening score and the FC of lPVT, rPTV and lNAc in TD children. Therefore, the FC between rPVT and lNAc was more reliable in assessing the degree of awakening ability in PNE children. This finding could help establish the evaluation index of PNE.

## Introduction

Primary nocturnal enuresis (PNE) refers to unconscious micturition behavior of children beyond 5 years old who can’t wake up at night to control micturition for at least two times a week. PNE affects about 8–20% for 5-year-old, and some persist into adulthood^1^. Frequent enuresis will seriously affect children’s physical and mental health, damage children's self-esteem, and even learning ability^2^. Up to now, the pathogenesis of PNE is still unknown, but most of the specialists believe it may be related to awakening dysfunction, insufficient secretion of antidiuretic hormone, unstable contraction of detrusor and other factors, among which awakening dysfunction is the prerequisite^3,4^. Only children with awakening dysfunction are unable to wake up at night with a full bladder, which is the basis for enuresis in children^5^. Both urorrhagia and bladder dysfunction cannot explain why children do not wake up to urinary stimulation. Normal adults and children can stimulate the brain to wake up when the bladder is full, even if they have a lot of urine at night or a poor ability to store urine in the bladder. Thus, awakening dysfunction is considered a prerequisite for PNE. Therefore, understanding the neural mechanism of awakening dysfunction is very important for the diagnosis and treatment of PNE.

The paraventricular thalamus (PVT) is a critical thalamic area for wakefulness and is the key brain region of the neural mechanism of PVT’s awakening regulating^6^. This study showed how sleep/wakefulness could occur through a coordinated shift in thalamic activity. This is by far the clearest and most definitive finding in the area of thalamic awakening regulating. Hu et al. found that the PVT play the role of awakening regulation by forming an excitatory synaptic functional connection with the nucleus accumbens (NAc). Activation of PVT-NAc pathway can induce the transition from sleep to wakefulness. On the contrary, inhibition of this pathway can reduce the level of awakening, indicating that the regulation effect of PVT on awakening depends on the PVT-NAc pathway^7^. This series of studies show that PVT, especially the PVT-NAc pathway, is very important for awakening regulation. In that way, do PNE children have something wrong with the PVT-NAc pathway leading to awakening dysfunction?

In order to study the function of the PVT-NAc pathway in PNE children, this study will adopt the analysis technique of resting-state functional magnetic resonance imaging (rs-fMRI) to study the function of separate brain regions of PVT and NAc and the functional connection between them. Previous studies on PNE by fMRI mainly focused on attention, working memory, et al. For example, a Go/NoGo task fMRI found response inhibition in children with PNE was associated with a relative lack of or delay in the maturation of prefrontal cortex circuitry^8^. In a working memory task fMRI study, decreased activation was found in the left cerebellum related to the dysfunction of working memory in PNE children^9^. There were also studies that combined genes and intelligence structures, or just aimed at the functional imaging changes in PNE^10–12^. In recent years, more and more researchers have focused on the fMRI changes based on awakening dysfunction. Zhu et al. used the methods of amplitude of low frequency fluctuations (ALFF), regional homogeneity (ReHo) and functional connectivity (FC) and found that left medial orbital superior frontal gyrus, left superior occipital gyrus and left medial superior frontal gyrus were correlated with awakening dysfunction. Abnormal brain activities were probably important neuropathological mechanisms of PNE in children^13^. Yu et al. used EEG-fMRI and found abnormal prefrontal and parietal thalamocortical FCs, accompanied by abnormal intrathalamic FCs among the motor, occipital, prefrontal and temporal subdivision of thalamus during light sleep, were related to abnormal sleep and enuresis in children with PNE^14^. The difference between this study and those studies was that this study focused on the key brain region of awakening (PVT), as well as NAc which was found to form an important pathway of awakening with PVT, so as to study the awakening dysfunction in children with PNE through the function and correlation of these two brain regions.

This study will study the spontaneous activity, degree centrality (DC)^15,16^, and functional connectivity^17^ to explore the brain mechanism of awakening dysfunction in children with primary nocturnal enuresis based on PVT-NAc neural pathway.

## Materials and methods

### Participants

PNE children were diagnosed by specialist clinic from Changzhou Children’s Hospital of Nantong University during January 2016 to December 2018 according to the Diagnostic and Statistical Manual of Mental Disorders, 5th Edition (DSM-V)^18^. The PNE group included 26 children (14 boys and 12 girls) aged 8.55 ± 1.60 years. These children must meet the following criteria: (1) older than 5 years old; (2) IQ > 80 (the Wechsler Intelligence Scale for Chinese Children-Revised)^19^; (3) having no urinary system disease; (4) right-handedness; (5) having no medical history related to neural and mental system; (6) bed-wetting at least twice a week and lasting more than 6 months; (7) bed-wetting only at night.

The typically developing group included 26 children (15 boys and 11 girls) aged 8.94 ± 1.59 years from an ordinary school in Changzhou. The criteria were the same as 1–5 of the PNE group. The study was approved by Changzhou Children’s Hospital of Nantong University (2014-001). Informed consent was obtained from the parent of each participant, and all the children agreed to participate in the study. The comparison of IQ between PNE and TD children was done by two-sample t-test and was found no statistical significant difference so as to exclude the influence of IQ on cognition (Table 1).

**Table 1:** Demographic and clinical data (x ± s).

	PNE (n = 26)	TD (n = 26)	T value	P value
Age	8.55 ± 1.60	8.94 ± 1.59	− 0.872	0.39
Sex (% male)	14 (54%)	15 (58%)	0.078	0.78
IQ	93.92 ± 12.88	97.73 ± 12.85	− 1.067	0.29
Bed-wetting frequency	4.31 ± 1.29	NA	NA	NA
Total voided volume	506.04 ± 97.33	380.42 ± 58.93	5.629	0.000*
Awakening score	5.38 ± 1.42	2.31 ± 0.93	0.256	0.000*

*P < 0.05. Total voided volume means the amount of urine remaining in the bladder. The chi-square test was used for P-value of Sex.

### Image acquisition

Siemens 1.5-T Magnetom Avanto scanner was used to collect the images. The children were all asked to lie supine with heads fixed for minimizing head motion. The children were instructed to close eyes and remain in a calm and awake status^20^. Rs-fMRI data were acquired to use an echo-planar imaging (EPI) sequence with the following parameters: repetition time (TR) = 2000 ms, echo time (TE) = 40 ms, 18 axial slices, flip angle = 90°, thickness/gap = 6.0/1.2 mm, field of view (FOV) = 240 × 240 mm, matrix = 64 × 64, 180 volumes (6 min). High-resolution T1-weighted three-dimensional (3D) images were acquired to cover the entire brain using the following parameters: TR = 414 ms, TE = 11 ms, flip angle = 90°, in-plane resolution = 256 × 256, FOV = 240 × 240 mm and thickness/gap = 5.0/1.5 mm^15^.

### Data analysis

The first ten volumes of rs-fMRI data were discarded to avoid transient signal changes and to allow the participants to adapt to the scanning noises. The data were then preprocessed using the data processing assistant for resting-state fMRI advanced edition (DPARSFA) V4.3 software package^21^. DPARSFA is a widely used rs-fMRI analytic tool based on the statistical parametric mapping (SPM8). The preprocessing included the following procedures: (1) slice timing correction; (2) head motion correction; (3) spatial normalization to a standard template (montreal neurological institute; MNI) and re-sampling (3 × 3 × 3 mm^3^); (4) removal of linear trend; (5) ALFF (after smooth) and DC (before smooth) calculation of the whole brain; (6) spatial smoothing with a Gaussian kernel of 6 mm full width at half maximum^15^.

### Scale for awakening score

The scale for awakening score is divided into 8°^10^: (1) Wakes up from the slightest noise or from turning on the light in the room; (2) Wakes up when called by name gently; (3) Wakes up when called by name loudly or sound of a bedside alarm clock; (4) Wakes upon shouting the name at the ear or upon gentle shaking; (5) Wakes up with loud noise and vigorous shaking; (6) Wakes up when physically stood up; (7) Wakes up when walked from the bed with support; (8) Doesn’t awaken, has to be carried out of bed.

### Statistical analysis

**Figure 1:**
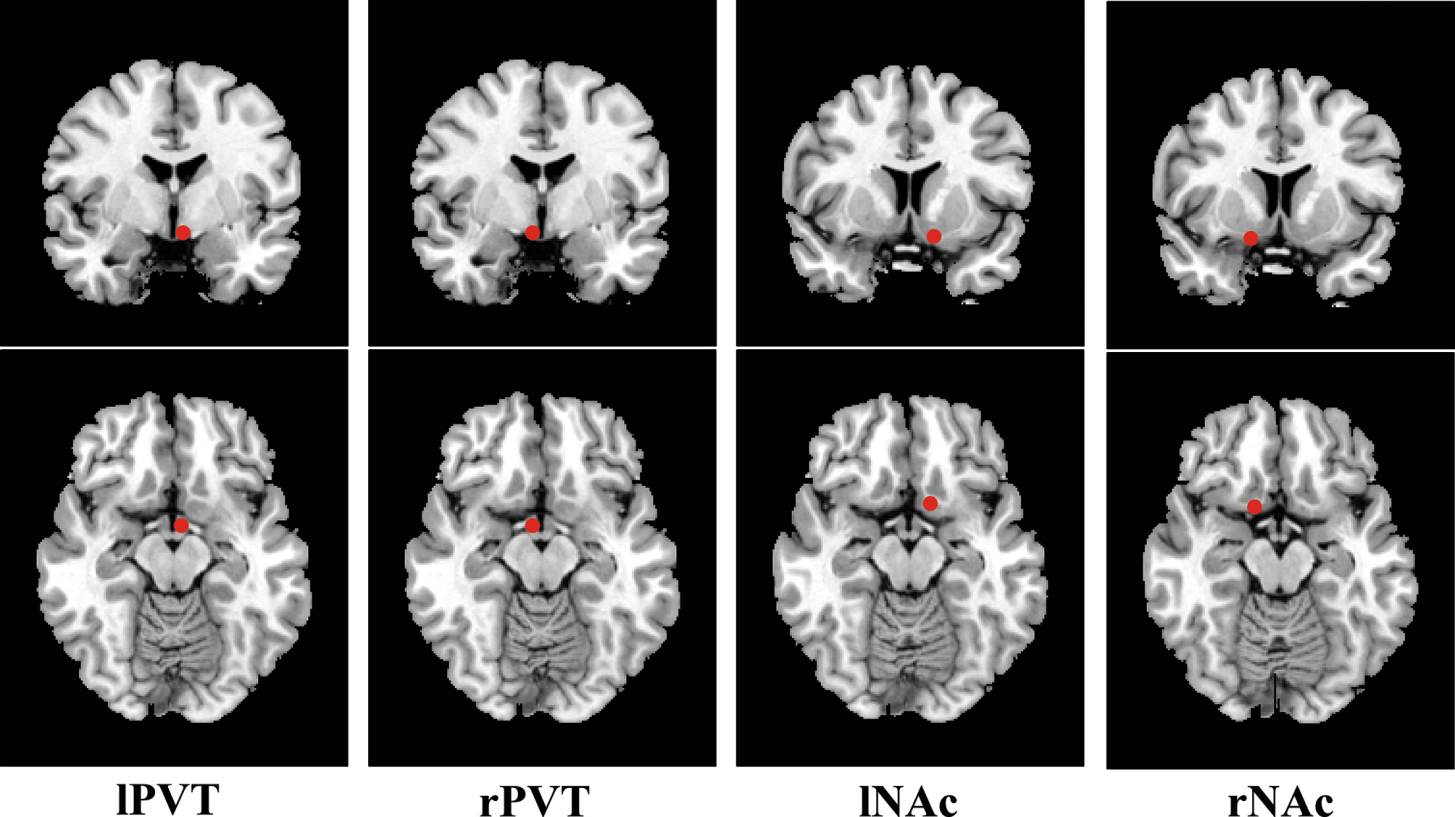
The coordinates of bilateral PVT and NAc.

Participants with head motion > 3 mm of translation or 3° of rotation in any direction were discarded and all the participants were eligible. The coordinates of lPVT (− 3, − 2, − 13), rPVT (3, − 2, − 3)^22^, lNAc (− 12, 9, − 14) and right NAc (rNAc) (14, 9, − 15)^23^ were selected as regions of interest (ROI) (Fig. 1), and the ALFF and DC values of ROI were extracted from the ALFF and DC values of the whole brain above. And the six groups of the FCs of these four coordinates were calculated.

Two-sample t-test was performed as a measure of the resting-state ALFF, DC and FC difference between PNE and TD children. The Alphasim correction was used in the study for the threshold of P < 0.05. The correlation between the awakening score and the fMRI indexes were analyzed by Pearson correlation analysis.

### Ethical statements

Informed consent was obtained from all individual participants included in the study.

### Ethics approval

We have obtained the ethical approval of the Institutional Review Board of Changzhou Children’s Hospital of Nantong University. All methods were performed in accordance with the relevant guidelines and regulations.

## Results

### ALFF and DC in bilateral PVT and NAc

**Figure 2:**
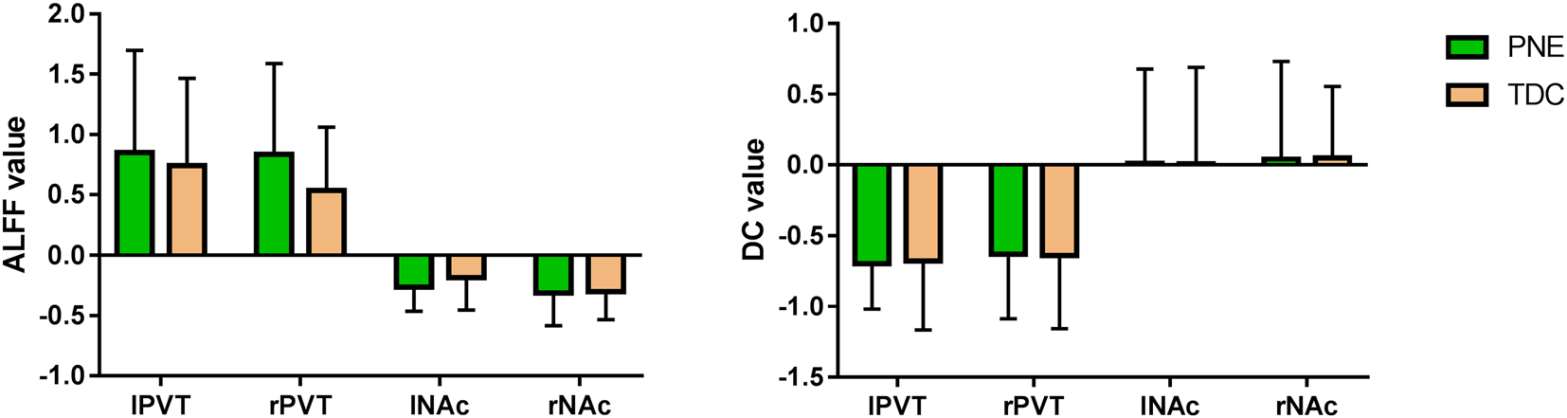
The comparison of ALFF and DC value of bilateral PVT and NAc.

There was no statistical significant difference in ALFF and DC between PNE and TD children in bilateral PVT and NAc (P > 0.05, Fig. 2 and Table 2).

**Table 2:** The comparison of ALFF and DC value of bilateral PVT and NAc.

	PNEALFF	TDALFF	t value	P value	PNEDC	TDDC	t value	P value
lPVT	0.85 ± 0.85	0.74 ± 0.72	0.496	0.622	− 0.70 ± 0.32	− 0.68 ± 0.49	− 0.152	0.880
rPVT	0.83 ± 0.76	0.53 ± 0.53	1.664	0.102	− 0.63 ± 0.46	− 0.64 ± 0.52	0.052	0.959
lNAc	− 0.26 ± 0.20	− 0.19 ± 0.27	− 1.166	0.249	0.01 ± 0.67	0.01 ± 0.68	0.027	0.979
rNAc	− 0.32 ± 0.27	− 0.30 ± 0.23	− 0.205	0.839	0.04 ± 0.69	0.05 ± 0.51	− 0.053	0.958

### FC between bilateral PVT and NAc

There was statistical significant difference of the comparison of the FC of the lPVT and the lNAc between PNE and TD children (P = 0.028) while other five FCs had no statistical significant difference (Table 3).

**Table 3:** The comparison of the FCs of bilateral PVT and NAc between PNE and TD children.

	PNE	TDC	t value	P value
lPVT-rPVT	1.55 ± 0.43	1.47 ± 0.33	0.713	0.479
lPVT-lNAc	0.03 ± 0.27	0.19 ± 0.24	− 2.261	0.028*
lPVT-rNAc	0.02 ± 0.30	0.17 ± 0.30	− 1.752	0.086
rPVT-lNAc	0.02 ± 0.29	0.17 ± 0.22	− 1.988	0.052
rPVT-rNAc	0.11 ± 0.30	0.24 ± 0.30	− 1.651	0.105
lNAc-rNAc	0.59 ± 0.28	0.73 ± 0.34	− 1.580	0.120

*Meant there was statistical significant difference.

### Correlation between awakening score and FC

The awakening score of PNE children (5.38 ± 1.42) was significantly higher than TD children (2.31 ± 0.93) (Higher the awakening score is, lower the degree of awakening ability is) (t = 0.256, P = 0.000).

**Figure 3:**
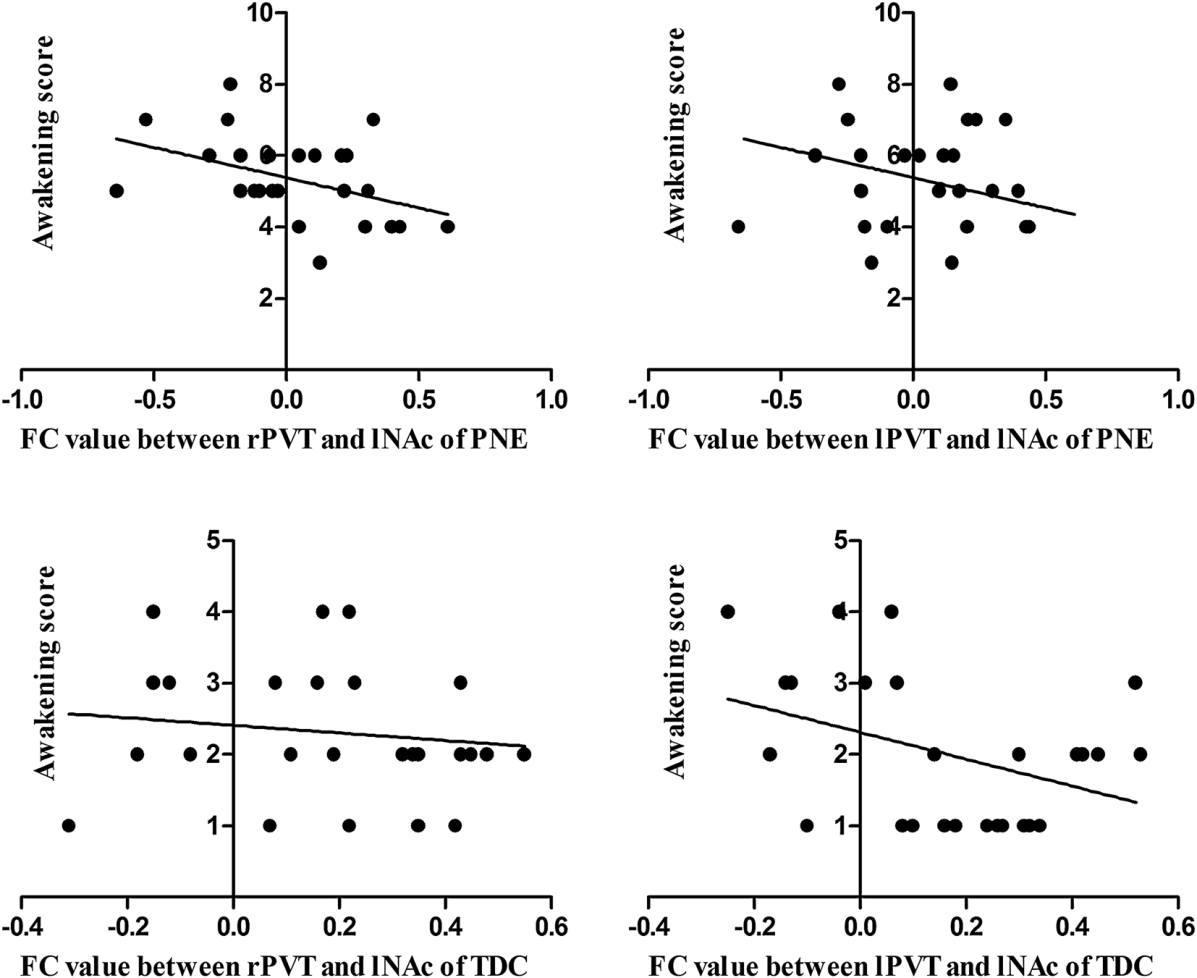
The correlation of the FC value between lNAc and bilateral PVT and awakening score of PNE and TD children.

The results showed that there was negative correlation between awakening score and the FC of rPVT and lNAc (r = − 0.430, P = 0.028), and no obvious correlation between awakening score and the FC of lPVT and lNAc in PNE children (r = − 0.370, P = 0.063). Meanwhile, there was both negative correlation between awakening score and the FC of lPVT (r = − 0.395, P = 0.046), rPTV (r = − 0.391, P = 0.048) and lNAc in TD children (Fig. 3).

## Discussion

This study found that there was no statistical significant difference in ALFF and DC between PNE and TD children in bilateral PVT and NAc. PVT is a non-specific nucleus group of the thalamus, which has the function of regulating excitability in a wide range of cortical areas, such as participating in awakening regulating^24,25^. PVT maintains high excitability during wakefulness, and its excitability increases sharply during the transition from sleep to awakening. Molecular biology experiments have proved that PVT plays a key role in maintaining wakefulness^26^. Then how does the PVT play the role of awakening regulation? The maintenance of wakefulness in the PVT depends on the PVT**-**NAc pathway. NAc has long been thought to be a key structure that mediates reward, addiction, and feeding activities which are based on awakening^27,28^. Luo et al. found NAc dopamine D1 receptor (D1R)-expressing neurons were essential for behavioral arousal. Optogenetic activation of NAc D1R neurons induces immediate transitions from non-rapid eye movement sleep to wakefulness, and chemogenetic stimulation prolongs arousal^29^. In this study, ALFF and DC methods were used to compare PNE and TD children at the four coordinates of bilateral PVT and NAc. ALFF is a method with high reliability and validity of fMRI reflecting the level of spontaneous brain activity^30^. DC reflects the central position of brain region based on the study of brain network^31^. This study found that there was no significant difference in ALFF and DC between the two groups, indicating that the PVT**-**NAc pathway did not play a direct role by a single brain region, but by the signal transduction between PVT and NAc to influence the awakening function. So we also used FC to study the functional connections between the two brain regions.

In this study, there was a statistical significant difference of the comparison of the FC of the lPVT and the lNAc between PNE and TD children while other five FCs had no statistical significant difference. The PVT-NAc projections and hypocretin neurons in the lateral hypothalamus to PVT glutamatergic neurons’ projections are the effector pathways for wakefulness control^6^. FC method reflects the synergistic consistency of two brain regions^17^. The results of this study showed that the FCs between lPVT and lNAc of PNE were lower than TD children, showing the pathway correlated with awakening were weaker. So children with PNE were harder to wake up and exhibited enuresis symptoms.

In combination with the findings of the FC studies, we analyzed the correlation between awakening score and the FC of bilateral PVT and lNAc. The other FCs didn’t find statistical significant differences between PNE and TD children which showed that they might not be the main FCs associated with awakening dysfunction of PNE. Therefore, we won’t discuss the other FCs correlated with awakening score here. Higher the awakening score is, lower the degree of awakening ability is. There was both negative correlation between awakening score and the FC of rPVT and lNAc in PNE and TD children, reflecting this group of FC had direct negative correlation with awakening score which indicated the FC could be the evaluation index of the degree of awakening ability. In TD children, there was negative correlation between awakening score and the FC of lPVT and lNAc, but none in PNE children. It indicated that this group of FC itself was correlated with awakening, but it was possible that PNE children might have dysfunction in their functional connections, resulting in an inability to reflect the association with awakening. Therefore, the FC between rPVT and lNAc was more reliable in assessing the degree of awakening ability in PNE children.

In conclusion, this study was based on the theory of “PVT is among the key brain regions implicated with awakening regulation and the regulation effect of PVT on awakening depends on the PVT-NAc pathway” which was reported in^6^. So we firstly compared ALFF and DC of four coordinates of bilateral PVT and NAc between PNE and TD children indicating that the awakening dysfunction of PNE children was not resulted by single brain region. Secondly, we compared the FC of the four brain regions and found there was a statistical significant difference of the comparison of FC of lPVT and lNAc between PNE and TD children. It may indicate a possible role of PVT-NAc pathway in awakening. At last, we analyzed the correlation between awakening score and the FC of bilateral PVT and lNAc and found there was both negative correlation between awakening score and the FC of rPVT and lNAc in PNE and TD children, indicating this group of FC could be the evaluation index of the degree of awakening ability. Otherwise, this study also had some limitations. The size of the samples was relatively small. And if the technique of polysomnography could be added in the assessment of awakening, the evaluation system would be more comprehensive. The data of rs-fMRI in wakefulness-controlling didn't be collected. And this research just compared the FC between PVT and NAC based on the premise of similar clinical manifestation of enuresis. And we’ll study the awakening function of PNE combining with more brain networks and regions.
